# Arterial Embolization with n-Butyl-Cyanoacrylate for a Large Gluteal Intramuscular Hemangioma: A Case Report

**DOI:** 10.3390/reports7040106

**Published:** 2024-11-26

**Authors:** Nicolas Papalexis, Michela Carta, Giuliano Peta, Simone Quarchioni, Maddalena Di Carlo, Marco Miceli, Giancarlo Facchini

**Affiliations:** Department of Diagnostic and Interventional Radiology, IRCCS Istituto Ortopedico Rizzoli, 40136 Bologna, Italy; michela.carta@ior.it (M.C.); giuliano.peta@ior.it (G.P.); simone.quarchioni@ior.it (S.Q.); maddalena.dicarlo@ior.it (M.D.C.); marco.miceli@ior.it (M.M.); giancarlo.facchini@ior.it (G.F.)

**Keywords:** vascular malformations, embolization, therapeutic, radiology, interventional, soft tissue tumors

## Abstract

**Background and Clinical Significance**: We wished to review the use of arterial embolization with n-butyl-cyanoacrylate (NBCA) to treat large high-flow vascular malformations due to its rapid polymerization and ability to permanently occlude large and small vessels. **Case Presentation**: A 52-year-old male presented with a two-year history of progressively worsening pain and swelling in the right gluteal area. Imaging techniques (color Doppler ultrasonography, CT, DSA, and MRI) were utilized for the diagnosis of a large high-flow intramuscular hemangioma. The mass displaced the surrounding tissues but showed no signs of lymphadenopathy or distant metastasis. The treatment involved targeting different arterial feeders over several sessions. Each procedure used NBCA–Lipiodol under fluoroscopic guidance, progressively reducing the malformation’s size and alleviating his symptoms. After the final embolization, the patient showed significant pain relief and a reduction in the size of the malformation, confirmed by follow-up imaging, demonstrating NBCA embolization’s effectiveness. The protocol’s safety and efficacy in this context are discussed. **Conclusions**: Arterial embolization with NBCA is a promising treatment for large high-flow vascular malformations, providing symptom relief and reductions in lesion size. While this case report highlights the procedure’s efficacy, further research is needed for a broader understanding of its long-term outcomes and potential complications.

## 1. Introduction and Clinical Significance

Vascular malformations are congenital anomalies in the blood vessels that can cause a range of symptoms depending on their size, location, and type [1,2]. They are classified into high-flow and low-flow lesions, with arteriovenous malformations (AVMs) being the most common high-flow lesions [3]. The incidence of vascular malformations varies, with the estimates for AVMs ranging from 1.2 to 1.5 per 10,000 live births [4,5]. Large vascular malformations can lead to pain, discomfort, and potential complications, such as bleeding and compression of the adjacent structures [6].

The current treatment for vascular malformations is often surgery; however, it carries a high recurrence rate [7]). Another effective option is sclerotherapy [8], but in our case, the predominance of the arterial flow over the venous flow led our team to choose embolization over other minimally invasive techniques.

Arterial embolization is a minimally invasive treatment option that has been successfully employed to treat a variety of musculoskeletal soft tissue tumors [9,10,11,12] and high-flow vascular malformations [13,14,15]. n-butyl-cyanoacrylate (NBCA) is a liquid adhesive agent and has the advantage of being very effective even in small volumes, occluding both small and large vessels permanently [16,17]. For this reason, NBCA was chosen in this case since in high-flow lesions, it provides durable and controlled occlusion [18].

This report aims to elucidate the application of NBCA in arterial embolization for the treatment of a large gluteal intramuscular hemangioma. Drawing on our clinical experiences and the procedural outcomes, we discuss the efficacy, safety, and potential of NBCA as a primary treatment modality for these challenging vascular anomalies.

## 2. Case Presentation

### 2.1. Medical History

A 52-year-old male presented to our department with a history of gradually worsening pain that started approximately 2 years prior and was associated with swelling and localized discomfort in the right gluteal area. At the beginning of symptom presentation, he underwent surgical excision, after which a diagnosis of intramuscular hemangioma was made. Shortly after the surgical excision, there was a rapid local recurrence of the lesion. The patient reported difficulty in performing daily activities, such as walking and climbing stairs, due to the increasing pain and size of the mass. The patient denied any constitutional symptoms, such as fever, night sweats, or unintentional weight loss.

Upon physical examination, a large, slightly tender, and pulsatile mass was palpable in the right gluteal area. The mass was firm and non-compressible. The overlying skin appeared normal, except for the surgical scar, without erythema, warmth, or ulceration. Distal pulses in the right lower extremity were intact.

Imaging studies, including color Doppler ultrasonography, contrast-enhanced computed tomography (CT), and MRI, were performed.

Color Doppler ultrasonography demonstrated increased vascularity and flow within the mass, suggesting the presence of a high-flow malformation.

A contrast-enhanced CT scan revealed a large, ill-defined, heterogeneously enhancing mass in the right gluteal and thigh regions, composed of many dysmorphic and extremely large arterial vessels. The mass had a total volume of 2628 cm^3^, as demonstrated in a pre-treatment MRI. The mass was located within the right great gluteus muscle and appeared to compress and displace surrounding soft tissue structures but did not infiltrate into any adjacent organs. No evidence of lymphadenopathy or distant metastasis was identified in the CT scan.

**Figure 1:**
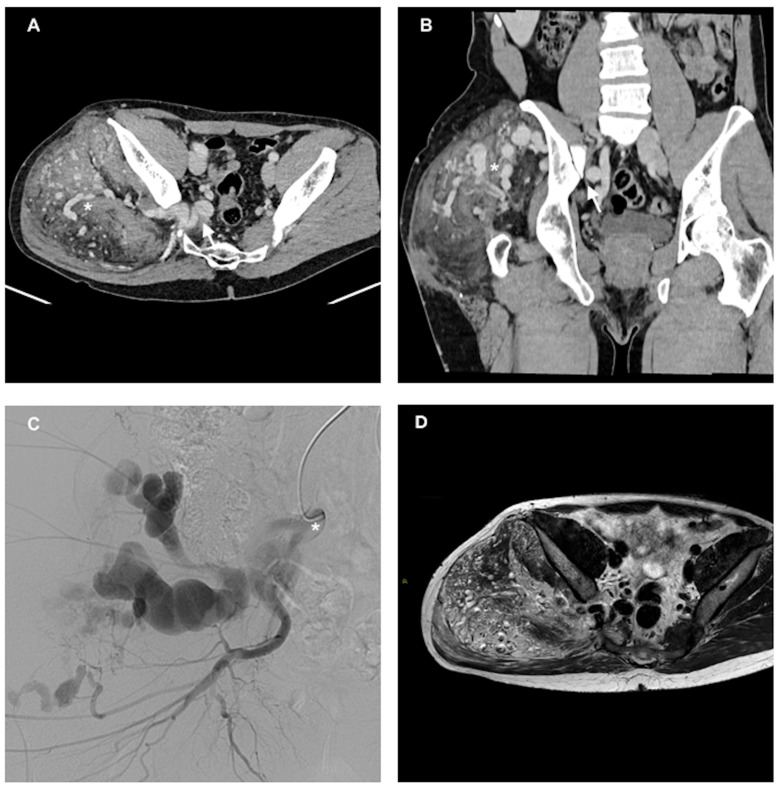
(**A**) Contrast-enhanced CT (axial) in the late arterial phase, demonstrating a large mass in the gluteal region, with many enlarged and tortuous feeding arterial vessels (asterisk), with a very tortuous and enlarged superior gluteal artery (arrow), and (**B**) the coronal view highlighting the cranio-caudal extension of the malformation and demonstrating enlarged arteries within the malformation (asterisk) and the enlarged internal iliac artery (arrow). (**C**) DSA performed with a 5 F Cobra catheter (asterisk) from the internal iliac artery demonstrating extremely enlarged superior and inferior gluteal arteries. (**D**) Pre-treatment MRI in an axial T2 non-saturated sequence showing the large lesion.

The malformation was supplied by multiple enlarged and tortuous arterial branches, arising mainly from the internal iliac artery (the superior and inferior gluteal arteries), and drained into the right internal iliac vein (Figure 1).

### 2.2. Treatment and Results

The patient underwent arterial embolization using NBCA on four separate occasions, with each procedure mainly targeting the superior and inferior gluteal arteries having new arterial feeders that were not present in the previous angiographies. The embolization was performed under local anesthesia and fluoroscopic guidance. An ultrasound-guided puncture of the contralateral common femoral artery was performed, a 5 French guiding catheter was then introduced through the femoral artery access, and a microcatheter was navigated into the feeding arteries. The first embolization targeted aberrant vessels originating from the internal iliac artery (the superior and inferior gluteal arteries) and the external iliac artery and its branches, including the deep circumflex iliac artery. A mixture of NBCA and Lipiodol (1:2) was slowly injected under continuous fluoroscopic monitoring, resulting in the occlusion of these abnormal vessels (Figure 1). Following the first embolization, there was a moderate reduction in the size of the malformation and an improvement in the patient’s symptoms. However, given the persistent aberrant arterial supply and the persistence of pain, a second embolization was performed eighteen months later. The aberrant vessels originated from the internal and external iliac arteries and their branches, including the superior and inferior gluteal arteries, the iliolumbar artery, and the lateral circumflex femoral artery. The same NBCA–Lipiodol mixture (1:2) was used to occlude these abnormal vessels.

Eight months after the second procedure, a third embolization was performed. The preliminary angiography showed partial revascularization from the superior and inferior gluteal arteries, which were, however, greatly reduced compared to in the previous angiographies. The same technique was used, and the abnormal vessels were successfully occluded with the NBCA–Lipiodol mixture (1:2).

**Figure 2:**
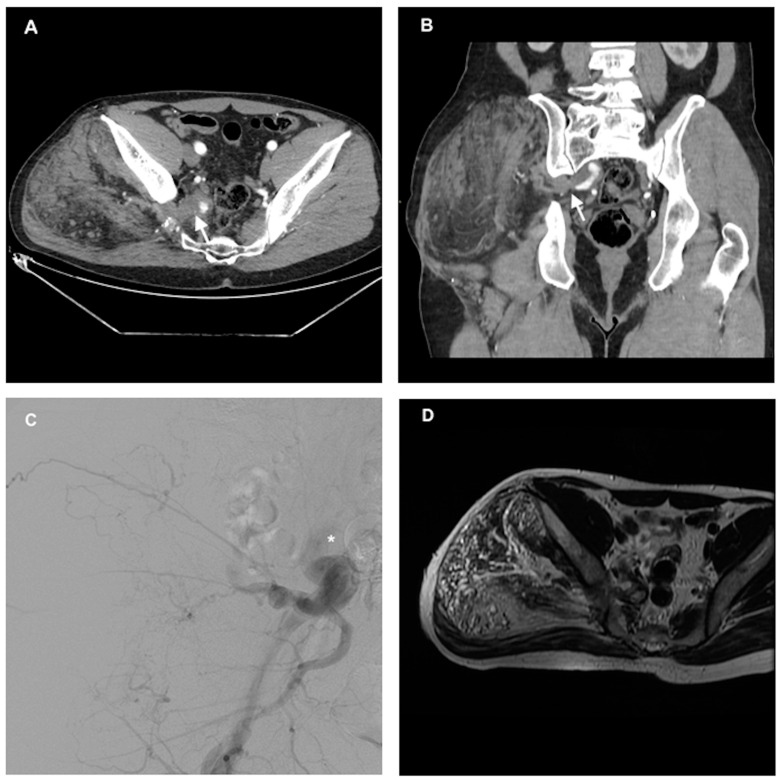
(**A**) Contrast-enhanced CT (axial) in the late arterial phase performed 6 months after the fourth (and last) embolization. The mass is considerably reduced in diameter, with some small arterial feeders, and thrombosis of the superior gluteal artery (arrow). (**B**) Coronal view demonstrating a great reduction in pathologic vascularization with a significant reduction in the caliber of the internal iliac artery (arrow). (**C**) DSA performed at the end of the fourth embolization procedure, with the tip of the catheter (Cobra 5 F) in the internal iliac artery (asterisk) demonstrating a great reduction in vascularization and a complete stop in the arterial flow in the superior gluteal artery. (**D**) Twelve-month follow-up MRI after the last embolization showing an axial T2 non-saturated sequence.

The fourth and last embolization was performed 4 months after to target the remaining vessels originating from the internal iliac artery. At this time, a complete stop in the blood flow from the superior gluteal artery was obtained (Figure 2).

After each embolization (four in total, over a period of three years), there was a progressive and significant reduction in the size of the malformation. The follow-up consisted of a non-contrast MRI every 6 months. Angio-CT was performed before the first treatment and after the third and fourth treatments to document the persistence of enlarged arteries that may have needed to be targeted by new treatment. After the fourth embolization, the patient’s pain and local discomfort had completely resolved. At the 12-month follow-up after the last embolization, angio-CT and non-contrast MRI demonstrated a significant reduction in the vascular malformation’s size, now with a total volume of 1521 cm^3^, with a great reduction in the size of the main feeding vessels and a reduction in the caliber of the internal iliac artery (Figure 2). Most notably, complete remission of symptoms was referred to by the patient; therefore, no further intervention was planned, and the patient was put on a yearly follow-up with MRI of the gluteal region.

## 3. Discussion

This report illustrates a unique case of a very large, high-flow intramuscular hemangioma with extremely large arterial feeders treated with four sessions of arterial embolization. After each treatment, there was a progressive reduction in the patient’s symptoms and the size of the lesion. However, multiple treatments were needed for this large vascular malformation due to the recanalization of previously embolized vessels.

In this case, MRI was the preferred imaging technique for assessing the lesion size and treatment response. Angio-CT was preferred for evaluating the vascular anatomy before or after treatment if vascular changes were suspected.

In the context of vascular malformations, early diagnosis and management are essential for preventing complications and improving a patient’s quality of life [19]. The treatment options for vascular malformations vary based on the lesion size, location, flow characteristics, and clinical presentation. In some cases, conservative management with compression garments, pain control, and monitoring may be appropriate [20]. However, for large or symptomatic lesions, more aggressive treatment options such as sclerotherapy, embolization, surgical resection, or a combination of these modalities may be necessary [3,13].

In this specific case, our team chose arterial embolization due to the significantly high arterial component of the malformation. The location was also favorable for embolization, as the feeding vessels originated from the gluteal artery, which typically lacks collateral connections with pelvic organs or other delicate structures. For low-flow and mostly venous malformations, percutaneous sclerotherapy should be considered first.

The choice of embolic agent is critical to the success of the arterial embolization procedure. NBCA has been shown to be particularly effective for treating high-flow lesions and is associated with low rates of recanalization and complications when it is used properly [21].

## 4. Conclusions

In conclusion, arterial embolization using NBCA can be a safe and effective treatment option, leading to significant improvement in patients’ symptoms. There are few articles available on the treatment of arterial vascular malformations. Studies with larger patient cohorts focusing on the treatment of extensive arterial vascular malformations could greatly enhance our understanding of the outcomes of this technique.

## Data Availability

The data presented in this study are available on request from the corresponding author due to privacy concerns.
